# MiRNAs from the Dlk1-Dio3 locus and miR-224/452 cluster contribute to glioblastoma tumor heterogeneity

**DOI:** 10.1038/s41598-024-58870-6

**Published:** 2024-04-13

**Authors:** Christopher M. Smith, Daniel Catchpoole, Gyorgy Hutvagner

**Affiliations:** 1 School of Biomedical Engineering, Faculty of Engineering and IT, University of Technology Sydney, Sydney, Australia; 2 Children’s Cancer Institute, Lowy Cancer Research Centre, UNSW Sydney, Sydney, NSW Australia; 3 School of Computer Sciences, Faculty of Engineering and IT, University of Technology Sydney, Sydney, NSW Australia; 4 The Tumour Bank, The Children’s Cancer Research Unit, Kids Research, The Children’s Hospital at Westmead, Westmead, NSW Australia

**Keywords:** Cancer, miRNAs

## Abstract

Glioblastoma is one of the most common and aggressive brain tumors and has seen few improvements in patient outcomes. Inter-tumor heterogeneity between tumors of different patients as well as intra-tumor heterogeneity of cells within the same tumor challenge the development of effective drugs. MiRNAs play an essential role throughout the developing brain and regulate many key genes involved in oncogenesis, yet their role in driving many of the processes underlying tumor heterogeneity remains unclear. In this study, we highlight miRNAs from the Dlk1-Dio3 and miR-224/452 clusters which may be expressed cell autonomously and have expression that is associated with cell state genes in glioblastoma, most prominently in neural progenitor-like and mesenchymal-like states respectively. These findings implicate these miRNA clusters as potential regulators of glioblastoma intra-tumoral heterogeneity and may serve as valuable biomarkers for cell state identification.

## Introduction

Glioblastoma is one of the most common and aggressive brain tumors and has seen few improvements in patient outcomes^1^. Inter-tumor heterogeneity between tumors of different patients as well as intra-tumor heterogeneity of cells within the same tumor challenge the development of effective drugs^2^. In glioblastoma, inter-tumor heterogeneity has been well characterized through large scale studies integrating gene expression data from The Cancer Genome Atlas (TCGA), initially identifying at least four key subtypes of glioblastoma named proneural (TCGA-PN), neural (TCGA-NE), classical (TCGA-CL), and mesenchymal (TCGA-MS), later revised to exclude the neural subtype^3,4^. More recently, intra-tumor heterogeneity in glioblastoma was characterized using single cell RNA-seq, which has provided a unified model for glioblastoma heterogeneity^5^. In this model they described four main cancer subpopulations, referred to as cell states, resembling cell types that exist during normal brain development. This included astrocyte-like (AC), neural-progenitor-like (NPC), oligodendrocyte-progenitor-like (OPC), and mesenchymal-like (MES) cell states. Furthermore, they highlighted that tumors were often comprised of multiple cell states, although not necessarily containing all states at once, and that the presence and relative frequencies of each cell state directly influenced TCGA subtype classifications at the population level.

MiRNAs play an essential role throughout the developing brain, contributing to cell fate specification and differentiation in many neural or glial stem/progenitor cells^6^. Extensive documentation of miRNA dysregulation in glioblastoma and their effects on key cancer pathways suggests they are also important in tumorigenesis^7^. Furthermore, miRNA expression profiles can significantly improve classification of tumors with TCGA subtypes, making them potential biomarkers and suggesting their activity is intrinsically linked to gene networks that drive each of these subtypes^8^. As research has now highlighted a direct link between TCGA subtypes and cell state compositions on a single cell level, this implies that miRNA expression is also associated with these cell states and may have important functions in cell state regulation^5^. Despite this, there is an absence of studies in the literature which aim to investigate miRNA expression in glioblastoma single cells and the role miRNAs play in the regulation of cell states. In this study, we highlight that miRNAs from the Dlk1-Dio3 and miR-224/452 clusters may be expressed cell autonomously and that their expression is associated with cell state genes in glioblastoma.

## Methods

### Single cell small RNA sequencing pre-processing and mapping

For the single cell small RNA sequencing reads, UMI sequences were removed prior to adapter removal and appended to the read headers. Adapters were then removed using cutadapt (v2.7) with a minimum overlap of 1 nt and maximum error rate of 0.1 between reads and adapter sequences^9^. After UMI and adapter removal, reads shorter than 15 nucleotides were excluded. To identify duplicated reads, reads were aligned to the human genome (hg38) using bowtie (v1.2.3) with the following parameters: -n 2 -e 120 -l 20 –best^10^. Human-aligned reads were subsequently deduplicated with umitools (v1.0.0) with default settings^11^.

Processed reads were aligned to miRbase (v22.1) annotated precursor miRNAs using miraligner (v3.4), with the following parameters: -sub 1 -trim 3 -add 3^12^. Reads which successfully aligned to a miRNA were also annotated with any variants to the miRbase defined mature sequence and converted to miRNA counts.

### Identification of cell subpopulations with miRNA expression

To identify glioblastoma cell subpopulations, miRNA expression was analyzed with the R package Seurat (v3.1.5)^13^. MiRNAs present in three or less cells, or cells with less than 1000 mapped reads, were excluded from analysis. For dimensionality reduction, Principal Component Analysis was used on the top 25% of variable features after centering and scaling the data. Cells were visualized using Uniform Manifold Approximation and Projection (UMAP). This was followed by cell clustering using Seurat’s FindNeighbors (k.param = 30) and FindClusters (resolution = 0.8) functions. Clustering of cells was stable across a range of parameters and was deemed acceptable due to the presence of cluster specific miRNA ‘markers’ highly expressed in one population and absent in the other. Seurat’s FindAllMarkers function was used for differential expression analysis, and only features with an adjusted *p*-value (*p*.adj) less than 0.05 and log_e_ fold-change larger than 1 were considered differentially expressed.

### Scoring for TCGA subtypes and cell states

Scores which reflect the expression of a set of genes (i.e. gene modules) were calculated using a method similar to that described in the Neftel et al. study^5^. Briefly, gene counts for all samples were converted to log_2_ transcripts-per-million (log_2_[TPM + 1]) and centered by deducting expression of each gene by its mean expression across all samples from the same dataset. Then for each sample, scores were calculated from the mean expression of the gene module minus the mean expression of a control gene set. For the control gene set, aggregate expression of each gene across all samples from the same dataset were used to organize genes into 30 expression bins. For each gene in the gene module, 100 genes were randomly selected from the same expression bin and placed in the control gene set.

The gene sets for the three TCGA subtypes were obtained from Wang et al.’s study^4^. Cell state gene sets were obtained from Neftel et al.’s study^5^.

## Results

### Expression of the Dlk1-Dio3 and miR-224/452 clusters characterize subpopulations of a glioblastoma primary culture.

**Figure 1:**
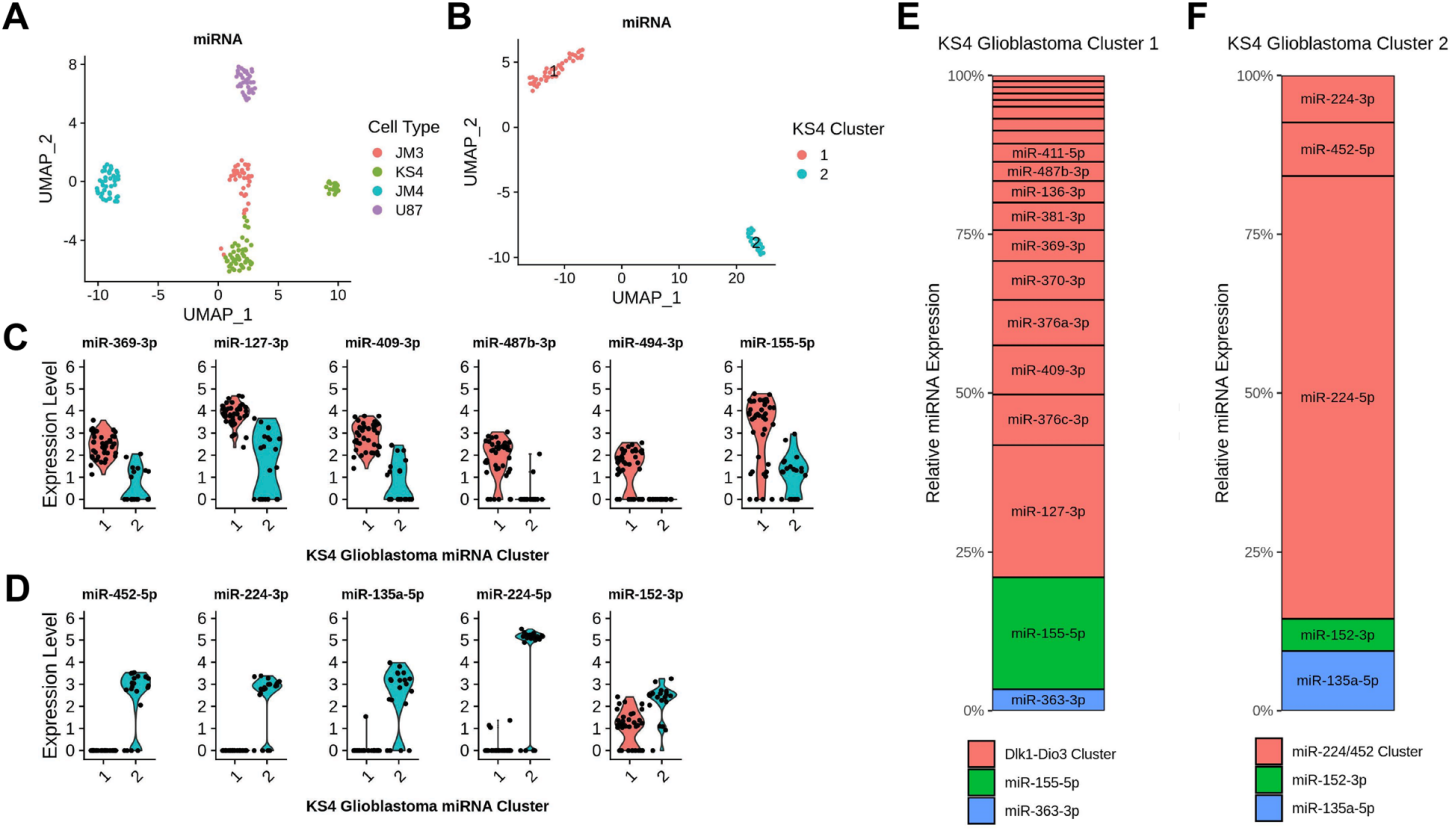
Identification of heterogenous glioblastoma cell subpopulations from miRNA expression. (**A**) UMAP plot of miRNA expression from four glioblastoma cell types. (**B**) UMAP plot for KS4 glioblastoma cells colored by cluster. Comparison of expression in select miRNAs that were differentially upregulated (adjusted *p*-value < 0.05) in KS4 cluster 1 (**C**) or KS4 cluster 2 (**D**). miRNA expression shown is in log_e_. Relative expression of the differentially expressed miRNAs for miRNAs upregulated in (**E**) KS4 Cluster 1 or (**F**) KS4 Cluster 2. miRNAs belonging to the same miRNA cluster were colored similarly.

To investigate if miRNA heterogeneity may contribute to cell state regulation or identity in glioblastoma, we analyzed publicly available single cell small RNA-seq data for 173 cells from 3 glioblastoma primary cultures and 1 glioblastoma cell line^14^. UMAP projections for miRNA expression (Fig. 1A) revealed that most cells co-localized with others from the same cell type with minimal evidence of common miRNA expression modules across different cell types. However, we observed that one of the glioblastoma primary cultures (KS4) formed two distinct groups, not associated with their batch number, that we hypothesized represent two subpopulations in different cell states (Fig. 1B and Supplementary Table 1).

Following this, we compared miRNA expression between the two KS4 cell clusters using differential expression analysis (Fig. 1C–D). In total, we identified 20 miRNAs which were significantly upregulated in KS4 cluster 1, and 5 miRNAs which were significantly upregulated in KS4 cluster 2 (*p*.adj < 0.05). Upon further investigation into the miRNAs upregulated in KS4 miRNA cluster 1, we noted that 18 of the 20 upregulated miRNAs originated from the same miRNA cluster hosted within the Dlk1-Dio3 gene locus on chromosome 14q32 (Fig. 1C and Supplementary Fig. 1). Three of the five upregulated miRNAs in cluster 2—miR-224-5p, miR-224-3p, miR-452-5p, were also from a single miRNA cluster on chromosome X (Fig. 1D). Out of the differentially expressed miRNAs identified between the two KS4 subpopulations, expression was dominated (> 75%) by the miRNAs from the Dlk1-Dio3 and miR-224/452 clusters (Fig. 1E–F).

### MiRNAs are associated with glioblastoma subtypes and cell state genes.

To predict the function of the upregulated miRNAs in the two KS4 clusters, we analyzed glioblastoma bulk RNA and miRNA expression data from the Clinical Proteomic Tumor Analysis Consortium (CPTAC) to identify any association between miRNA expression and marker genes for glioblastoma subtypes or known cell states. Previous work demonstrated that glioblastoma subtypes are indicative of the most dominant cell states within a tumor, with the proneural subtype (TCGA-PN) typically enriched with cells in a neural-progenitor-like (NPC) or oligodendrocyte-progenitor-like (OPC) state, mesenchymal subtype (TCGA-MS) enriched with cells in mesenchymal-like (MES) state, and classical subtype (TCGA-CL) enriched with cells in an astrocyte-like (AC) state^5^. Therefore, we reasoned that if the miRNA pathway had a role in regulating cell states, then it may be possible to identify miRNAs with strong associations to the genes typically upregulated in these cell states using population level data.

**Figure 2:**
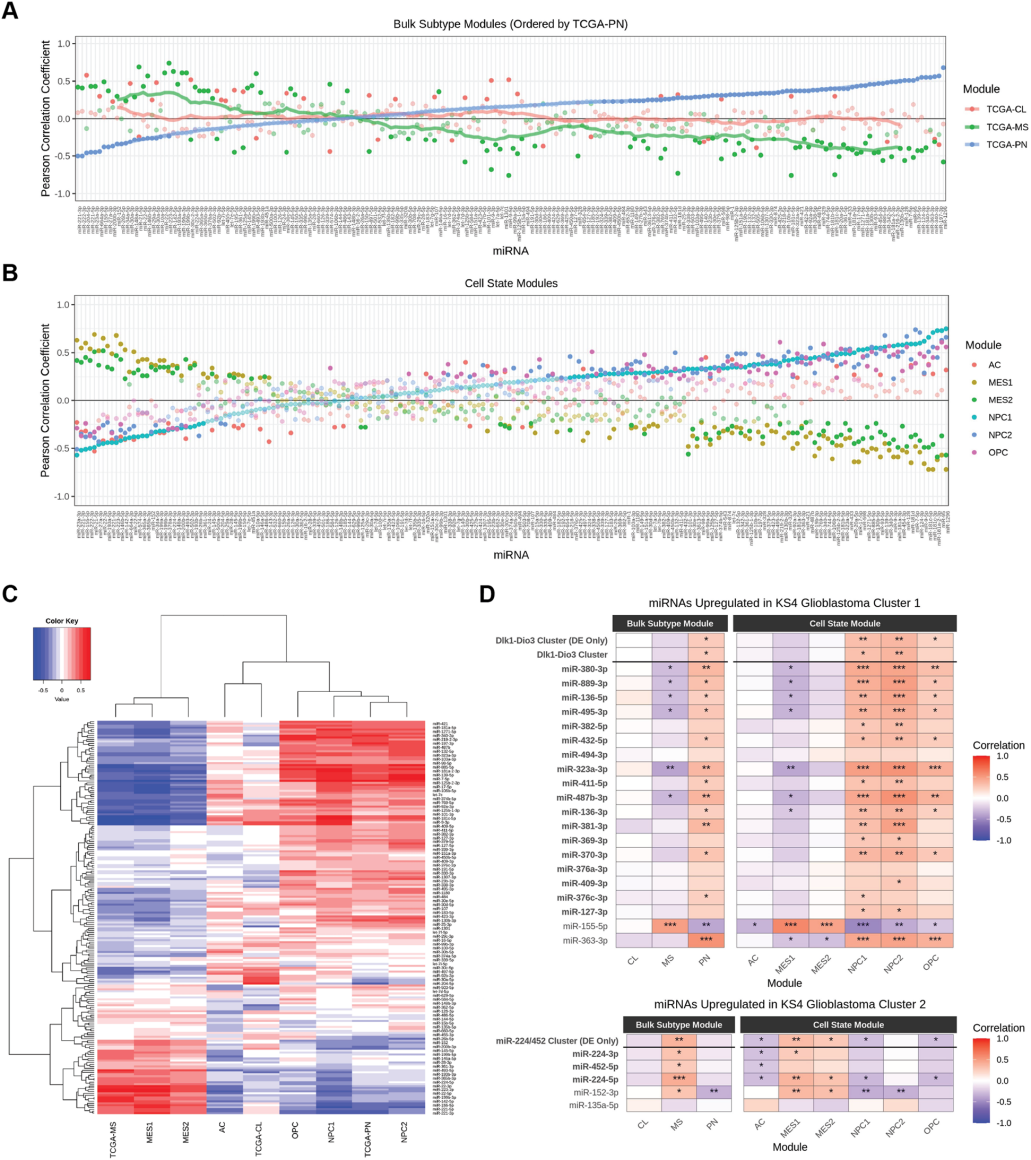
Expression of miRNAs correlate with TCGA subtypes and cell state modules. Pearson correlation for the 200 highest expressed miRNAs and (**A**) TCGA subtype or (**B**) cell state module scores. miRNAs are ordered by ascending Pearson correlation coefficients for the Proneural (TCGA-PN) subtype. Non-significant Pearson coefficients are shown as transparent dots. (**C**) Hierarchical clustering of Pearson correlation values between each miRNA’s expression and corresponding gene module score. (**D**) Pearson correlation of miRNAs upregulated in KS4 cluster 1 (top) or (bottom) KS4 cluster 2 with each TCGA subtype or cell state score. miRNAs belonging to the Dlk1-Dio3 miRNA cluster or miR-224/452 cluster are shown in bold, as well as combined expression of all miRNAs from each cluster (All) or only miRNAs differentially expressed between KS4 cells (DE Only). P-values are shown as asterisks—*: 0.005 <  = *p*-value < 0.05; **: 0.0005 <  = p-value < 0.005; ***: 0.00005 <  = *p*-value < 0.0005. CL: Classical; MS: Mesenchymal, PN: Proneural; AC: Astrocyte-like; MES: Mesenchymal-like; NPC: Neural-progenitor-like; OPC: Oligodendrocyte-progenitor-like.

To detect potential associations between miRNAs and TCGA subtypes or cell states we scored tumors by their mean expression of marker genes associated with subtype or cell state^5^. Tumors were typically associated with at most one TCGA glioblastoma subtype and scores for each TCGA subtype were negatively correlated (Pearson’s R = − 0.47 to − 0.31; Supplementary Fig. 2). Out of the top 200 expressed miRNAs measured across all glioblastoma tumors, 146 miRNAs were significantly correlated with at least one subtype score (*p*.adj < 0.05; Fig. 2A). The strongest correlations of miRNA expression and subtype scores were with the TCGA-PN and TCGA-MS subtypes.

Cell states were defined according to the four cell states described by Neftel et al., and included six gene modules in total, one module associated with each of the AC and OPC states and two modules associated with each of the NPC and MES states^5^. Cell state module scores were correlated against expression of each miRNA (Fig. 2B). The highest Pearson correlation values were observed with the NPC1, NPC2, MES1, and MES2 cell states. Strong anti-correlation patterns with similar magnitudes were observed between the NPC1/NPC2 states and MES1/MES2 states across most miRNAs. A high number of miRNAs had matching correlation signs between NPC1, NPC2, OPC, and AC cell states, although correlation was generally weaker with the OPC and AC cell states.

The correlation of scores and miRNA expression was similar between the TCGA-Proneural subtype and NPC1/NPC2 states, as well as the TCGA-MS subtype and MES1/MES2 states (Fig. 2C). This was consistent with Neftel et al.’s previous work suggesting these subtypes were predominantly composed of cells exhibiting these respective states^5^. A weaker association was observed between the TCGA-CL subtype and each cell state, with the highest association to the AC state (R = 0.34), possibly reflecting this subtype’s mixed population of states.

### The Dlk1-Dio3 and miR-224/452 clusters are associated with glioblastoma cell states.

Focusing on the differentially expressed miRNAs identified between the two KS4 glioblastoma subpopulations, most miRNAs that were co-upregulated in one of these subpopulations had matching signs of correlation coefficients for each cell state (Fig. 2D). For example, 16/20 (*p*.adj < 0.05) miRNAs upregulated in KS4 cluster 1 were positively correlated with the NPC1 cell state score, 16/20 (*p*.adj < 0.05) with the NPC2 score, and 10/20 (*p*.adj < 0.05) with the OPC score. Conversely there was a negative correlation with 8/20 (*p*.adj < 0.05) miRNAs and the MES1 scores. All 18 of the miRNAs from the Dlk1-Dio3 locus had a positive correlation coefficient with the NPC scores, however some were weakly correlated or not statistically significant. Only one miRNA, miR-155-5p, appeared to contradict the miRNAs in this group, having a positive correlation with MES1 scores and negative correlations with NPC1, NPC2, and OPC scores. Out of the five miRNAs upregulated in KS4 cluster 2 (Fig. 2D), three were positively correlated with the MES1 scores and two with the MES2 scores, as well as two negatively correlated with NPC1 scores, and one with NPC2 and OPC scores. All three miRNAs from the miR-224/452 cluster negatively correlated with AC cell state scores. We also considered that an aggregated expression of miRNAs from each cluster (Fig. 2D), including all miRNAs belonging to this cluster (All) or only those differentially expressed between the two KS4 glioblastoma subpopulation (DE Only), may provide a stronger association to these cell states than any individual miRNA. We found for both measurements of the Dlk1-Dio3 cluster expression (Fig. 2D), correlation was similarly positive with the NPC1, NPC2, and OPC states, although not significant with the OPC state for the differentially expressed miRNAs. No significant negative correlation was shown between the aggregated Dlk1-Dio3 miRNA expression and the AC, MES1, and MES2 scores. For the aggregated miR-224/452 cluster miRNA expression, significant positive correlation was observed with the MES1 and MES2 states, and negative correlation with the NPC1, OPC, and AC state scores (both All and DE Only). The evidence indicated that expression of select miRNAs, such as miR-323a-3p and miR-224-5p, may be on par or better than an aggregate expression of the clusters for distinguishing between cell states.

Finally, we noted that the cell state scores were generally consistent with the bulk subtype scores (Fig. 2D), with the miRNAs upregulated in KS4 Cluster 1 generally positively correlating with the TCGA-PN subtype score and miRNAs from KS4 cluster 2 positively correlating with the TCGA-MS subtype score. The combined evidence of cell autonomous regulation of these two miRNA clusters as well as their association with specific cell states highlights a novel form of intra-tumor heterogeneity in glioblastoma and implicates them as potential regulators of glioblastoma cell states.

### The Dlk1-Dio3 cluster lncRNA MEG3 is associated with the neural progenitor-like and oligodendrocyte progenitor-like cell states in single cells.

Previous studies suggest that miRNAs from the Dlk1-Dio3 locus are co-transcribed from a common primary transcript with 3 long non-coding RNAs—MEG3, MEG8, and MEG9^15^. To investigate expression of this locus in single cells we analyzed single cell RNA-seq data from four studies. Although single cell RNA-seq does not capture miRNAs, we hypothesized that expression of these long non-coding RNAs would be strongly correlated with miRNA expression from this locus and would be potential marker genes for Dlk1-Dio3 miRNA expression in single cells.

Firstly, to determine if the long non-coding RNAs MEG3, MEG8, and MEG9 could predict expression of miRNAs from the Dlk1-Dio3 locus, we used paired bulk RNA and miRNA expression data for glioblastoma tumors from the CPTAC dataset to determine the correlation of an aggregated expression of Dlk1-Dio3 miRNAs with each gene in the RNA dataset. There was a high correlation between the combined Dlk1-Dio3 miRNA expression and the non-coding RNAs MEG8 (Pearson’s r = 0.71), MEG3 (Pearson’s r = 0.67), and MEG9 (Pearson’s r = 0.65), as well as with RTL1 (Pearson’s r = 0.65), all of which are encoded from this locus (Supplementary Table 2). Excluding genes which code for miRNAs or snoRNAs, these four were the most positively correlated across the glioblastoma tumors, indicating their expression corresponded well with Dlk1-Dio3 miRNA expression. This provided strong support for their use as markers to infer miRNA expression from this locus when only RNA-seq data is available.

To investigate if the Dlk1-Dio3 marker genes were associated with any cell states we utilized single cell RNA-seq data from 4 different studies, which included 50 glioblastomas (45 unique tumors) and 16,269 cells after filtering^5,16–18^. Of the Dlk1-Dio3 markers, only MEG3 was detectable in a high number of cells so we focused on this gene.

**Figure 3:**
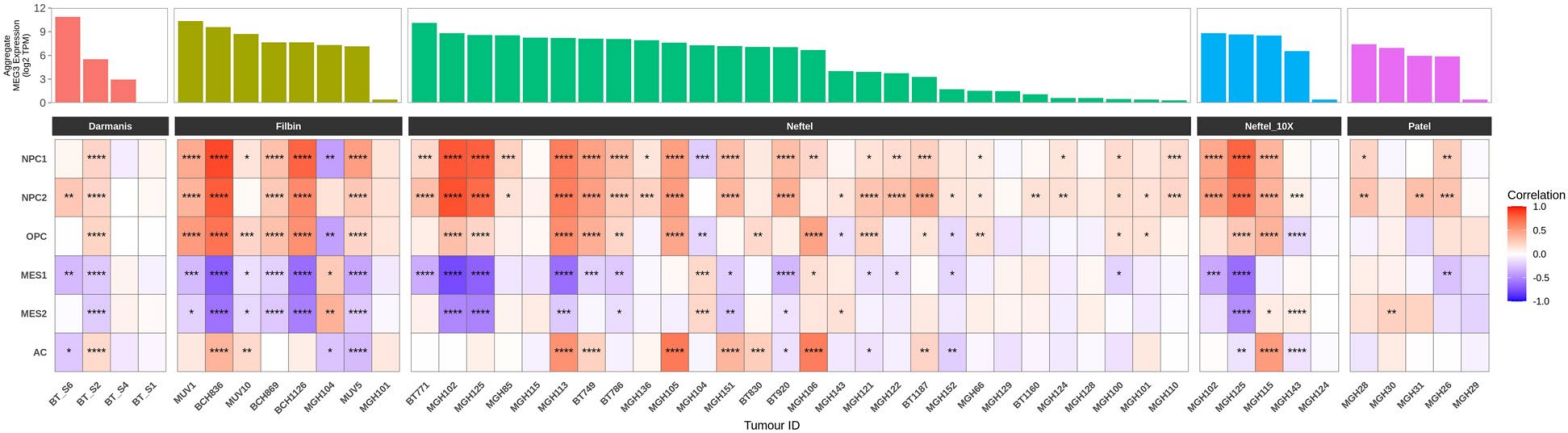
MEG3 is associated with glioblastoma cell states. MEG3 expression (top) and Pearson correlation with cell state module scores (bottom) in glioblastoma cells from different patients. P-value significance is shown as asterisks. *: 0.005 <  = *p*-value < 0.05; **: 0.0005 <  = *p*-value < 0.005; ***: 0.00005 <  = *p*-value < 0.0005; ****: 0.000005 <  = *p*-value < 0.00005. AC: Astrocyte-like; MES: Mesenchymal-like; NPC: Neural-progenitor-like; OPC: Oligodendrocyte-progenitor-like.

Glioblastoma cells were assigned a score for each cell state using the same methodology described previously. We calculated the Pearson correlation of cell state module scores and MEG3 expression (log_2_[TPM + 1]) for all cells in each dataset. In most tumors (38/50), MEG3 expression was positively correlated with at least one of the NPC cell state scores, and many were also positively correlated with the OPC scores (22/50; Fig. 3). Tumors with a positive correlation of MEG3 expression and NPC scores generally had a corresponding negative correlation with the MES1 and/or MES2 scores. Although correlations between MEG3 and cell state scores were typically weak to moderate in strength (R < 0.7), the results were consistent with our previous findings for the Dlk1-Dio3 miRNAs in the population-level data.

## Discussion

In this study we identified miRNAs from the Dlk1-Dio3 and miR-224/452 clusters which distinguished two subpopulations of cells in a primary glioblastoma culture and show that both miRNA clusters are associated with different glioblastoma cell states. Our results suggest miRNA expression from the Dlk1-Dio3 locus, as well as the putatively co-expressed long non-coding RNA MEG3, is positively associated with the NPC cell state and negatively associated with the MES state. The opposite was true with the miR-224/452 cluster where it was positively associated with the MES cell state but negatively associated with the NPC cell state. However, cause-and-effect is difficult to establish via association and it remains to be seen if any of these miRNAs are capable of driving or maintaining cancer cell states, or if their expression simply changes in response to other, more critical genes. Even in the absence of a functional role, the miRNA highlighted in this study may hold value as biomarkers for cell states once the field evolves towards clinical strategies which factor intra-tumoral heterogeneity.

Recently, a lot of attention has focused on accurately classifying glioblastoma subtypes using smaller gene panels which may be more feasible for use in a clinical setting. The initial Verhaark classification identified a total gene set of 840 genes for classifying classical, mesenchymal, neural, proneural signatures, later reduced to a 48 gene classifier that retained 90.91% accuracy^3,19^. A 50 gene signature was later defined for IDH-wild type glioblastoma which excluded the neural subtype^4^. A later study by Fu et al. demonstrated 83.7% accuracy in classifying tumors into proneural or mesenchymal subtypes utilizing a smaller set of 26 genes^20^. None of these studies utilized miRNA expression for classification and our results suggest that a combination of both RNA and miRNA expression may further enhance this accuracy.

A key question is whether defining cell states with RNA or miRNA expression will identify the same cell states or if examining each expression modality will lead to the detection of different subpopulations. In a study with hepatocellular carcinoma cells, the authors used hierarchical clustering to identify three subpopulations of cells with RNA expression but found only two with miRNA expression^21^. There was significant overlap between two of the RNA expression-based subpopulations and the miRNA subpopulations, however the third was not identifiable in the miRNA expression data. This suggests that it cannot always be assumed that there is concordance between RNA-based cell states and miRNA expression. Consequently, if the miRNAs examined in this study, including those from the Dlk1-Dio3 and miR-224/452 clusters, regulate cell subpopulations in a manner that is different to the cell states used in this study, then it is less likely that we would observe any strong association with them. However, our study indicates RNA and miRNA expression may classify similar populations.

MiRNAs such as miR-9-3p, miR-27a, and miR-23a, have previously been identified as markers for discriminating TCGA-PN and TCGA-MS subtypes^22^. Results from our study were consistent with this work, as these three miRNAs had some of the strongest correlation coefficients with these subtypes compared to other miRNAs, supporting the validity of these findings. How well these observations translate to single cell level miRNA expression and their association with cell states remains to be confirmed.

We highlighted an inverse relationship between RNA expression of NPC/OPC genes and MES genes. This observation was also evident in bulk miRNA expression data when comparing NPC/OPC and MES scores and suggests miRNA expression is highly responsive to cell states and likely incorporated into the same gene networks. Although we have preliminary evidence that the two subpopulations of KS4 cells are most strongly associated with the NPC and MES cell states, further research which pairs miRNA sequencing with a more direct way of identifying biologically relevant cell states is critical. Furthermore, studies will need to be scaled up to include multiple tumors to determine if heterogenous expression of miRNAs from the Dlk1-Dio3 and miR-224/452 clusters are a common feature in glioblastoma or an isolated case.

In conclusion, our study is the first to implicate the Dlk1-Dio3 and miR-224/452 miRNA clusters as potential regulators of glioblastoma intra-tumoral heterogeneity and may serve as valuable biomarkers for cell state identification.

### Supplementary Information

Supplementary Information.

## Data Availability

Raw single cell small RNA sequencing reads were obtained from the Gene Expression Omnibus (GEO) database under accession id GSE81287. Raw or normalized gene counts and metadata from glioblastoma single cell RNA sequencing was obtained the Gene Expression Omnibus (GEO) database under accession ids GSE84465 (Darmanis dataset), GSE131928 (Neftel and Neftel_10X datasets), GSE102130 (Filbin dataset), and GSE57872 (Patel dataset)^5,16–18^. Pre-mapped read counts from bulk miRNA and RNA sequencing of glioblastomas were obtained from the GDC Data Portal under project ID CPTAC-3.
