# Supplementary material for: MiRNAs from the Dlk1-Dio3 locus and miR-224/452 cluster contribute to glioblastoma tumor heterogeneity
Source: Sci Rep. 2024 Apr 13;14:8570. doi: 10.1038/s41598-024-58870-6 (PMC11014907; doi:10.1038/s41598-024-58870-6)
Supplement: Supplementary file 1 — Supplementary Information. [file 41598_2024_58870_MOESM1_ESM.docx]

**Supplementary Information**

## MiRNAs from the Dlk1-Dio3 locus and miR-224/452 cluster contribute to glioblastoma tumor heterogeneity.

Christopher M. Smith^1,2^, Daniel Catchpoole^3,4^, Gyorgy Hutvagner^1*^

^1^School of Biomedical Engineering, Faculty of Engineering and IT, University of Technology Sydney

^2^Children’s Cancer Institute, Lowy Cancer Research Centre, UNSW Sydney, NSW, 2052, Australia

^3^School of Computer Sciences, Faculty of Engineering and IT, University of Technology Sydney, PO Box 123, Broadway, 2007, NSW, Australia.

^4^The Tumour Bank, The Children’s Cancer Research Unit, Kids Research, The Children’s Hospital at Westmead, Locked Bag 4001, Westmead, NSW, 2145, Australia

*Correspondence to be sent: gyorgy.hutvagner@uts.edu.au


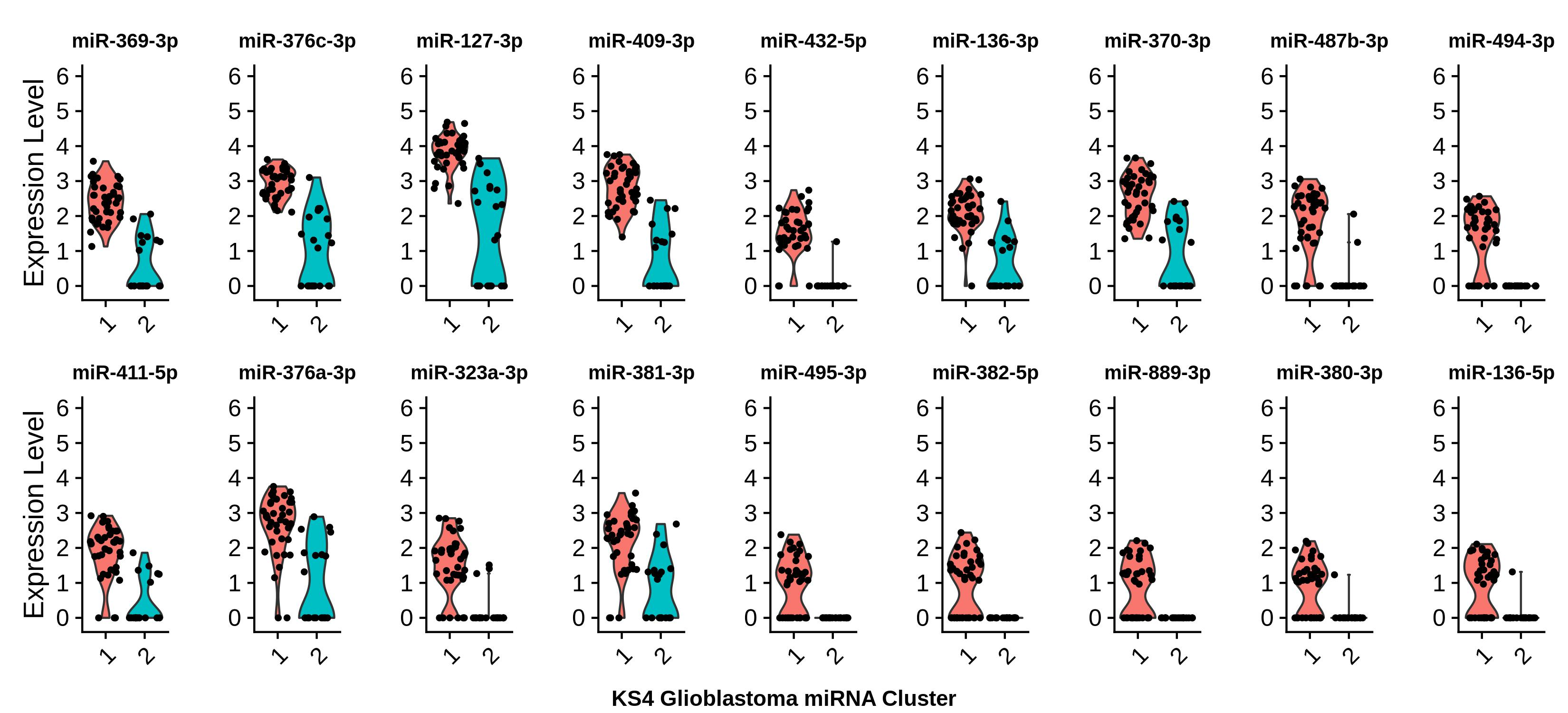


**Supplementary Figure 1.** Dlk1-Dio3 locus derived miRNAs upregulated in KS4 glioblastoma clusters 1 (red) and 2 (blue). Only miRNAs with adjusted p-values less than 0.05 were included. miRNA expression shown is in log_e_.


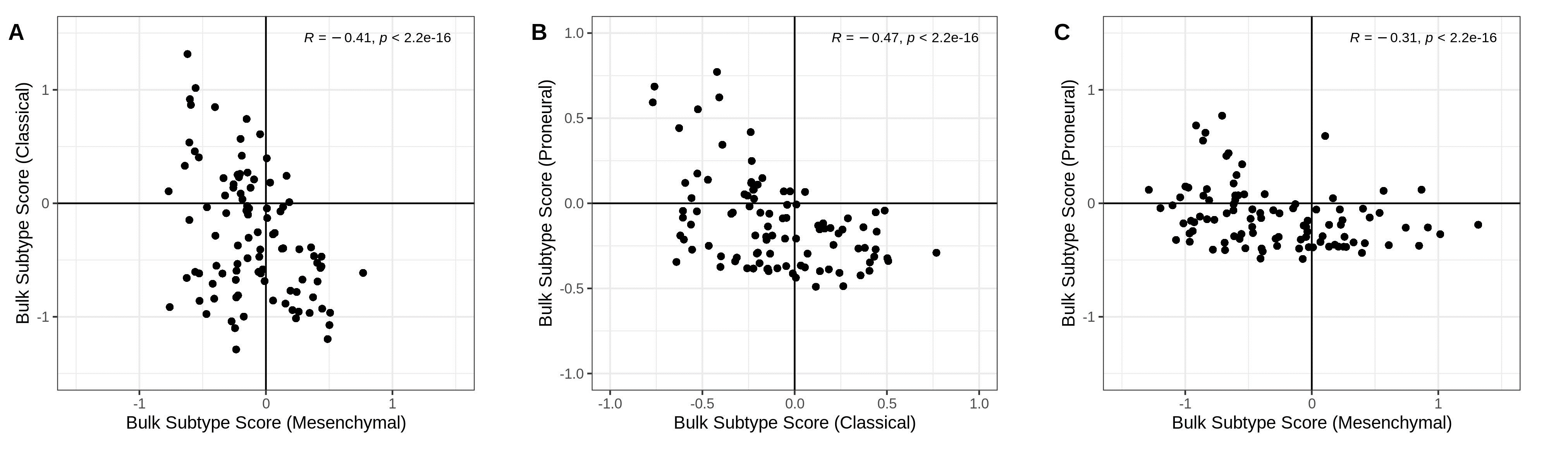


**Supplementary Figure 2.** Comparison of each pair of TCGA subtype scores in glioblastoma tumors. Scores and Pearson correlation are shown for **(A)** Mesenchymal vs Classical, **(B)** Classical vs Proneural, and **(C)** Mesenchymal vs Proneural.

| **Sample** | **Cell_Type** | **cell_label** | **AvgSpotLen** | **KS4 miRNA Cluster** | **batch** |
| --- | --- | --- | --- | --- | --- |
| SRR3495547 | KS4 | KS4 | 43 | 1 | 1 |
| SRR3495548 | KS4 | KS4 | 43 | 2 | 1 |
| SRR3495549 | KS4 | KS4 | 43 | 1 | 1 |
| SRR3495550 | KS4 | KS4 | 43 | 1 | 1 |
| SRR3495551 | KS4 | KS4 | 43 | 1 | 1 |
| SRR3495552 | KS4 | KS4 | 43 | 1 | 1 |
| SRR3495553 | KS4 | KS4 | 43 | 1 | 1 |
| SRR3495554 | KS4 | KS4 | 43 | 1 | 1 |
| SRR3495555 | KS4 | KS4 | 43 | 2 | 1 |
| SRR3495556 | KS4 | KS4 | 43 | 1 | 1 |
| SRR3495557 | KS4 | KS4 | 43 | 1 | 1 |
| SRR3495558 | KS4 | KS4 | 43 | 1 | 1 |
| SRR3495559 | KS4 | KS4 | 43 | 2 | 1 |
| SRR3495560 | KS4 | KS4 | 43 | 1 | 1 |
| SRR3495561 | KS4 | KS4 | 43 | 1 | 1 |
| SRR3495562 | KS4 | KS4 | 43 | 1 | 1 |
| SRR3495563 | KS4 | KS4 | 43 | 2 | 1 |
| SRR3495564 | KS4 | KS4 | 43 | 2 | 1 |
| SRR3495565 | KS4 | KS4 | 43 | 1 | 1 |
| SRR3495566 | KS4 | KS4 | 43 | 2 | 1 |
| SRR3495567 | KS4 | KS4 | 43 | 1 | 1 |
| SRR3495568 | KS4 | KS4 | 43 | 1 | 1 |
| SRR3495570 | KS4 | KS4 | 43 | 1 | 1 |
| SRR3495571 | KS4 | KS4_D | 43 | 2 | 1 |
| SRR3495572 | KS4 | KS4_D | 43 | 1 | 1 |
| SRR3495573 | KS4 | KS4_D | 43 | 1 | 1 |
| SRR3495574 | KS4 | KS4_D | 43 | 2 | 1 |
| SRR3495575 | KS4 | KS4_D | 43 | 1 | 1 |
| SRR3495576 | KS4 | KS4_D | 43 | 2 | 1 |
| SRR3495577 | KS4 | KS4_D | 43 | 2 | 1 |
| SRR3495578 | KS4 | KS4_D | 43 | 2 | 1 |
| SRR3495579 | KS4 | KS4_D | 43 | 1 | 1 |
| SRR3495580 | KS4 | KS4_D | 43 | 1 | 1 |
| SRR3495581 | KS4 | KS4_D | 43 | 2 | 1 |
| SRR3495582 | KS4 | KS4_D | 43 | 2 | 1 |
| SRR3495583 | KS4 | KS4_D | 43 | 1 | 1 |
| SRR3495584 | KS4 | KS4_D | 43 | 1 | 1 |
| SRR3495585 | KS4 | KS4_D | 43 | 2 | 1 |
| SRR3495586 | KS4 | KS4_D | 43 | 1 | 1 |
| SRR3495587 | KS4 | KS4_D | 43 | 1 | 1 |
| SRR3495588 | KS4 | KS4_D | 43 | 1 | 1 |
| SRR3495589 | KS4 | KS4_D | 43 | 2 | 1 |
| SRR3495590 | KS4 | KS4_D | 43 | 1 | 1 |
| SRR3495591 | KS4 | KS4_D | 43 | 1 | 1 |
| SRR3495592 | KS4 | KS4_D | 43 | 2 | 1 |
| SRR3495593 | KS4 | KS4_D | 43 | 1 | 1 |
| SRR3495594 | KS4 | KS4_D | 43 | 1 | 1 |
| SRR3495815 | KS4 | KS4 | 51 | 1 | 2 |
| SRR3495816 | KS4 | KS4 | 51 | 1 | 2 |
| SRR3495817 | KS4 | KS4 | 51 | 1 | 2 |
| SRR3495818 | KS4 | KS4 | 51 | 2 | 2 |
| SRR3495819 | KS4 | KS4 | 51 | 2 | 2 |
| SRR3495820 | KS4 | KS4 | 51 | 1 | 2 |
| SRR3495821 | KS4 | KS4 | 51 | 1 | 2 |
| SRR3495822 | KS4 | KS4 | 51 | 1 | 2 |
| SRR3495823 | KS4 | KS4 | 51 | 1 | 2 |
| SRR3495824 | KS4 | KS4 | 51 | 1 | 2 |
| SRR3495825 | KS4 | KS4 | 51 | 2 | 2 |
| SRR3495826 | KS4 | KS4 | 51 | 1 | 2 |
| SRR3495827 | KS4 | KS4 | 51 | 2 | 2 |

**Supplementary Table 1.** Metadata associated with KS4 cells including miRNA cluster and batch number.

| **Gene** | **Pearson’s Correlation Coefficient** | **P-value** | **Gene in Dlk1-Dio3 locus** |
| --- | --- | --- | --- |
| MEG8 | 0.71 | 1.92E-16 | Yes |
| MEG3 | 0.67 | 7.01E-14 | Yes |
| MEG9 | 0.65 | 4.88E-13 | Yes |
| RTL1 | 0.65 | 5.55E-13 | Yes |
| EIF2S1 | 0.54 | 1.18E-08 | No |
| ATP5MJ | 0.52 | 4.07E-08 | No |
| PSMC1 | 0.50 | 1.27E-07 | No |
| COA8 | 0.48 | 4.20E-07 | No |
| SLC39A9 | 0.48 | 6.62E-07 | No |
| UBR7 | 0.48 | 5.17E-07 | No |
| BAG5 | 0.47 | 1.21E-06 | No |
| CINP | 0.47 | 8.29E-07 | No |
| EIF5 | 0.47 | 9.53E-07 | No |
| VTI1B | 0.46 | 1.64E-06 | No |
| AREL1 | 0.45 | 3.49E-06 | No |
| FAM131B-AS1 | 0.45 | 3.60E-06 | No |
| PPP2R3C | 0.45 | 2.94E-06 | No |
| PPP4R3A | 0.45 | 3.07E-06 | No |
| DRAXIN | 0.43 | 1.29E-05 | No |
| DYNC1H1 | 0.43 | 9.72E-06 | No |

**Supplementary Table 2.** Top 20 genes with the highest correlation to the combined (mean) expression of Dlk1-Dio3 miRNAs, excluding genes encoding for miRNAs (i.e primary or precursor miRNAs) and snoRNAs.
